# Cavin1; a Regulator of Lung Function and Macrophage Phenotype

**DOI:** 10.1371/journal.pone.0062045

**Published:** 2013-04-25

**Authors:** Praveen Govender, Freddy Romero, Dilip Shah, Jesus Paez, Shi-Ying Ding, Libin Liu, Adam Gower, Elizabeth Baez, Sherif Shawky Aly, Paul Pilch, Ross Summer

**Affiliations:** 1 The Pulmonary Center, Boston University School of Medicine, Boston, Massachusetts, United States of America; 2 Center of Translational Medicine and Division of Pulmonary and Critical Care Medicine, Thomas Jefferson University, Philadelphia, Pennsylvania, United States of America; 3 Department of Biochemistry, Boston University School of Medicine, Boston, Massachusetts, United States of America; Louisiana State University Health Sciences Center, United States of America

## Abstract

Caveolae are cell membrane invaginations that are highly abundant in adipose tissue, endothelial cells and the lung. The formation of caveolae is dependent on the expression of various structural proteins that serve as scaffolding for these membrane invaginations. Cavin1 is a newly identified structural protein whose deficiency in mice leads to loss of caveolae formation and to development of a lipodystrophic phenotype. In this study, we sought to investigate the functional role of Cavin1 in the lung. Cavin1 deficient mice possessed dramatically altered distal lung morphology and exhibited significant physiological alterations, notably, increased lung elastance. The changes in distal lung architecture were associated with hypercellularity and the accumulation of lung macrophages. The increases in lung macrophages occurred without changes to circulating numbers of mononuclear cells and without evidence for increased proliferation. However, the increases in lung macrophages were associated with higher levels of macrophage chemotactic factors CXCL2 and CCL2 in BAL fluid from Cavin1−/− mice suggesting a possible mechanism by which these cells accumulate. In addition, lung macrophages from Cavin1−/− mice were larger and displayed measurable differences in gene expression when compared to macrophages from wild-type mice. Interestingly, macrophages were also increased in adipose tissue but not in liver, kidney or skeletal muscle from Cavin1−/− mice, and similar tissue specificity for macrophage accumulation was observed in lungs and adipose tissue from Caveolin1−/− mice. In conclusion, this study demonstrates an important role for Cavin1 in lung homeostasis and suggests that caveolae structural proteins are necessary for regulating macrophage number and phenotype in the lung.

## Introduction

The branched structure of the lung, culminating in alveolae, provides the large surface area needed for efficient gas exchange in this tissue. The maximization of the area involved in gas exchange extends to the cellular level, in that type I epithelial cells and endothelial cells are highly enriched in cell surface invaginations, resulting in a functional enhancement of cell surface capacity [1]. These surface invaginations, also called caveolae, are not exclusive to the lung. Most notably, caveolae are found in the highest concentrations in adipose tissue, and are present in many other tissues at lower levels [2]. The importance of caveolae in these tissues is related less to their role in increasing surface area and more to their ability to regulate diverse biological processes such as glucose and lipid homeostasis, clathrin-independent pinocytosis, and a wide-range of intracellular trafficking and signaling events.

The formation of caveolae is dependent on the production of various structural proteins that serve as scaffolding for these membrane invaginations. Genetic mutations in select structural proteins lead to the absence of caveolae formation and development of complex pathological phenotypes [3]–[7]. The most recognized abnormalities associated with deficiency in these proteins are metabolic derangements, and in mice and humans, these include hyperglycemia, insulin resistance and lipodystrophy [8]–[11]. However, it is also increasingly apparent from knockout studies that caveolae carry out many other important functions separate from their role in regulating metabolism [2], [12].

In particular, caveolae have been shown to play a critical role in orchestrating lung homeostasis. For example, targeted deletion of either caveolin 1 or 2 proteins leads to a wide range of lung abnormalities, including increased deposition of extracellular matrix, dysregulated cell growth and proliferation, and impaired pulmonary vascular function [3], [4]. Although the mechanisms leading to these diverse changes are poorly understood, changes to epithelial, endothelial and fibroblast function are well-documented.

Recently, a new group of caveolar structural proteins, the cavins, have been identified. This family of proteins, like the caveolins, is essential for the biogenesis and function of caveolae [13]. Included in this family is a 43-kDa cytoplasmic protein known as PTRF (Polymerase I and Transcript Release Factor) or Cavin1 (also known in past as Cav-P60) [14]–[17]. Like the caveolins, Cavin1 is highly expressed in multiple tissues, including the lung, but murine knockout studies have described only lipodystrophic phenotypes [9]. Based on this limited knowledge of Cavin1, the focus of this investigation was to understand the functional role of this newly recognized caveolar protein in lung homeostasis.

## Materials and Methods

### Ethics Statement

All experiments were carried out in accordance with the recommendations in the Guide for the Care and Use of Laboratory Animals of the National Institutes of Health and study protocols were approved by Boston University’s Institutional Animal Care and Use Committee (IACUC number AN-14683).

### Mice

Studies were performed on 2-month-old female mice. Cavin1 knockout (Cavin1−/−) mice on a C57Bl/6 background were generated as previously described [9]. Cavin1−/− mice and controls were obtained by breeding Cavin1+/−. Cavin1−/+ mice yield small litters but surviving pups, like Cavin1−/− mice, behave normally and have normal life expectancies when followed to 1 year. We have previously reported that adult Cavin1−/− mice are leaner when compared to their litter mate controls [9]. Caveolin-1 knockout (Cav1−/−) mice and age-matched controls were purchased from The Jackson Laboratory (Bar Harbor, ME).

### Lung Physiology Measurements

In an anesthetized mouse, an 18G tracheal cannula was inserted into a surgically exposed trachea and ligated tightly. The mouse was then placed on the FlexiVent mechanical ventilator (Scireq Scientific Respiratory Equipment, Montreal, Quebec), and ventilated at 300 breaths/min with positive-end expiratory pressure set at 3 cmH_2_O. Baseline measurement of airway resistance and lung elastance was measured as previously described [18].

### Western Blot Analysis

Western blot was performed using 20 µg of protein isolated from homogenized lung tissue or macrophage cell lines (MH-S or RAW 264.7 cells). Protein samples were separated by SDS-PAGE and electrophoretically transferred to polyvinylidene difluoride (PVDF) membranes. Membranes were incubated in phosphate-buffered saline with 0.1% Tween 20 containing 10% nonfat dry milk for 1 h at room temperature, followed by incubation with primary rabbit antibody directed against murine Cavin1 (21^st^ Century Biochemicals; Marlboro, MA), CD45 (Abcam, Cambridge, MA), GM-CSF (Abcam, Cambridge, MA) or β-actin (Cell Signaling Technology; Beverly, MA). Horseradish peroxidase-conjugated secondary antibodies (Sigma-Aldrich; St Louis, MO) and an enhanced chemiluminescence (ECL) substrate kit (PerkinElmer Life Sciences, Waltham, MA) were used for detection.

### Immunohistochemistry

Formalin-fixed tissues were prepared for frozen or paraffin sectioning using standard techniques. For paraffin-embedded tissues, wax was removed by solvents (Histo-Clear; National Diagnostics, Atlanta, GA) and tissues were re-hydrated in graded alcohols prior to antibody staining. For some antibodies, antigen retrieval was required using a heated citric acid solution (Target Retrieval Solution; Dako, Carpinteria, CA). For all sections, hydrogen peroxide in methanol (3%, 15 min, 22°C) was used to quench endogenous peroxidases and background staining was reduced by blocking with 2% BSA. Primary antibodies used in these studies included rabbit polyclonal anti-Cavin1 (21^st^ Century Biochemicals; Marlboro, MA), rat anti-mouse CD45 (BD Biosciences no. 550539), rat anti-mouse Mac-3 (BD Biosciences no. 550292), biotinylated rat anti-mouse NK1.1 (BioLegend clone PK136), biotinylated rat anti-mouse B-220 (BD Biosciences no. 553086), biotinylated rat anti-mouse CD-3 (BioLegend no. 100303), or purified rat anti-mouse GR-1 (R&D systems no. MAB1037). All primary antibodies were diluted in ranges from 1∶100 to 1∶500 and applied for 1 hour at RT. Biotinylated antibodies were detected using an ABC kit (Vector Laboratories) followed by addition of 3,39-diaminobenzadine. Cavin1, CD-45, NK 1.1, Mac-3, and GR-1 were detected using an anti-rabbit (Cavin1) or anti-rat secondary antibody kit (Vector Laboratories Burlingame, CA) before exposure to 3,39-diaminobenzadine. PCNA (Invitrogen Grand Island, NY) and TUNEL (R&D Systems Minneapolis, MN) staining were performed using kits according to manufacturer’s protocol.

### Tissue Morphometry

Quantification of leukocytes was performed after antibody staining in tissue sections. Evaluation was performed by two independent investigators, one of who was blinded to the study group. Mac3, CD4, NK1.1, B220 and Gr-1+ cells were quantified in three histological sections from each mouse. Because of structural changes in lungs of Cavin1−/− mice, the quantification of Mac3+ cells was performed by two methods. First, we calculated the number of cells per high power field. Second, we evaluated the number of Mac3+ cells per alveolus. For the latter analysis, 300 alveoli were counted per mouse in each group.

### Lung DNA Content and Wet-to-dry Ratios

DNA content and lung wet-to-dry ratios were determined per published protocols [19], [20].

### ELISA

Thermo Scientific Nunc Maxisorp plates were coated with primary antibody against CCL2 or CX3CL1 (R&D Systems Minneapolis, MN) overnight at RT and then washed with PBS and 0.5% Tween 20. Plates were blocked with 1% BSA in PBS for 1 h, followed by addition of samples for 2 h incubation at RT. After washing, biotinylated secondary antibody was applied for 2 h, followed by streptavidin-HRP conjugate diluted at 1∶200 with the blocking reagent. Reaction was measured using endpoint spectrometry after being developed with 0.01% tetramethylbenzidine dissolved in DMSO and 0.5% hydrogen peroxide.

### Real-time Quantitative PCR

RNA was isolated from homogenized lungs using RNeasy Plus Mini kit (Qiagen; Valencia, CA). Real-time quantitative PCR was performed on cDNA converted from isolated RNA using the Reverse Transcription System kit (Promega; Madison, WI). The CFX96 Touch™ Real-Time PCR Detection System (BioRad; Hercules, CA) was used to measure gene expression for granulocyte-monocyte colony-stimulating factor (GM-CSF), surfactant protein C (SPC), Clara cell 10 (CC10), T1-α, CD31 and CD45 (Taqman gene expression probes, Applied Biosystems; Carlsbad, CA). The 18S rRNA transcript was used to normalize RNA concentration for each sample.

### Flow Cytometry and Cell Sorting

Single-cell suspensions of enzyme-digested lung tissue were obtained using published protocols [21]. Cell suspensions were immunostained with rat anti-mouse CD45, F4/80 and CD11c, and in separate studies for CD11c and CD11b (BD Biosciences, Franklin Lakes, NJ). Isotype controls were used in all studies. Antibody stained cells were subjected to flow cytometry analysis. Propidium iodide was used to exclude dead cells from analysis.

### Bronchoalveolar Lavage and Peripheral Blood Cell Counts

Bronchoalveolar lavage was performed using previously described methods [22]. The total number of peripheral blood leukocytes was calculated using a hemocytometer and differential counts were performed on blood smears after Diff-Quik staining.

### Microarray analysis

All procedures were performed at the Boston University Microarray Resource Facility. Briefly, the total RNA was isolated using QIAGEN’s RNeasy kit (Qiagen, Valencia, CA) and sample integrity was verified using RNA 6000 Pico Assay RNA chips run in Agilent 2100 Bioanalyzer (Agilent Technologies, Palo Alto, CA). Total RNA (5 ng) was reverse transcribed using Ovation Pico WTA System V2 (Nugen, San Carlos, California). The obtained SPIA-amplified cDNA was purified using Agencourt RNA clean XP Purification Beads and fragmented (5 ng) and labeled with biotin using the Encore Biotin Module (NuGEN, San Carlos, California). SPIA-amplified cDNA and fragmented cDNA quality controls were carried out by running an mRNA Pico assay in the Agilent 2100 Bioanalyzer.

The labeled, fragmented DNA was hybridized to the Mouse Gene 1.0 ST Array (Affymetrix, Santa Clara, CA) for 18 hours in a GeneChip Hybridization oven 640 at 45°C with rotation (60 rpm). The hybridized samples were washed and stained using an Affymetrix fluidics station 450. After staining, microarrays were immediately scanned using an Affymetrix GeneArray Scanner 3000 7G Plus (Affymetrix, Santa Clara, CA).

Raw Affymetrix CEL files were normalized to produce Entrez Gene-identifier-specific expression values using the implementation of the Robust Multiarray Average (RMA) [23] in the affy package [24] in the Bioconductor software suite (version 2.10.0) [25] and an Entrez Gene-specific probeset mapping from BrainArray (version 14.0.0) [26], [27]. Genes were considered expressed if their mean expression across all samples exceeded the median expression of all genes across all samples. Differential gene expression between wild type and Cavin 1−/− samples was assessed using the empirical Bayes (moderated) *t* test from the limma package [28]. All microarray analyses were performed using the R environment for statistical computing (version 2.12.0) [29], [30].

### Gene Set Enrichment Analysis (GSEA)

GSEA was used to identify biological terms, pathways and processes that were coordinately up- or down-regulated with respect to various comparisons [31]. A list of all Entrez Gene identifiers interrogated by the array was ranked according to the moderated *t* statistic computed between the wild-type and Cavin 1−/− samples, and this list was then used to perform a pre-ranked GSEA analysis using the Entrez Gene versions of the Biocarta, KEGG, Reactome, and Gene Ontology (GO) gene sets obtained from the Molecular Signatures Database (MSigDB), version 3.0 [32].

### Statistical Analysis

Statistics were performed using GraphPad Prism 5.0 software. Comparisons between groups were performed using a 2-tailed unpaired Student *t* test or Mann Whitney U test. To control for multiple comparisons in GSEA analysis, Benjamini-HochbergFalse Discovery Rate (FDR) correction was applied. Significance of correlation was determined using the Pearson’s test.

## Results

**Figure 1:**
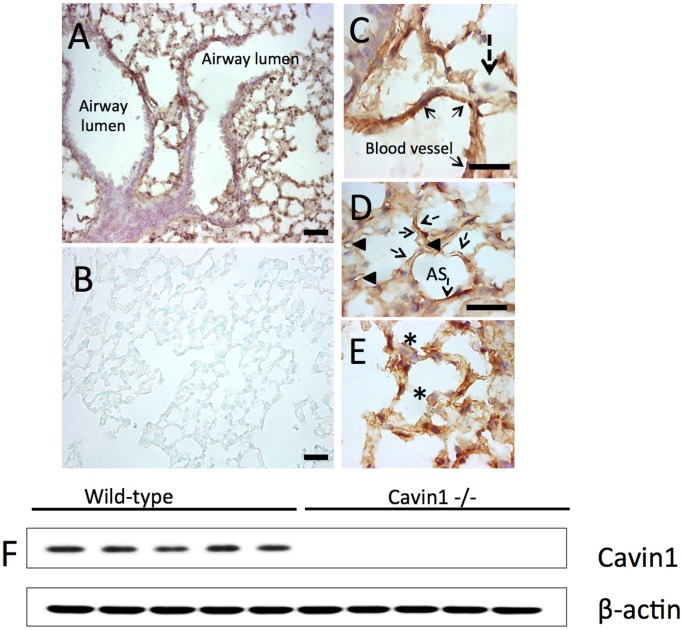
Immunohistochemistry staining of lung for Cavin1. A) Cavin1 staining is absent from proximal lung (purple) but localizes to distal murine lung (brown color). B) As expected, Cavin1 staining is absent from the lungs of Cavin1−/− mice. C-E) High power images of wild-type lung demonstrate Cavin1 expression on blood vessel endothelium (solid arrows, C), capillaries (arrowheads, D), type-I pneumocytes (broken arrows, D), alveolar macrophages (asterisk, E). Staining is absent from type-II epithelial cells in distal airspaces (broken arrow, C). F) The specificity of Cavin1 antibody was demonstrated by Western blot analysis.

Immunohistochemistry was performed on frozen sections in order to localize Cavin1 in lung tissue. Staining demonstrated Cavin1 to be absent from proximal airway epithelium but abundantly expressed on large and small blood vessel endothelium and throughout distal airspaces (brown stain, Fig. 1). High power images of distal airspaces further localized Cavin1 protein to alveolar macrophages and type I epithelial cells, but not to cuboidal type II epithelial cells (Fig. 1C-E). As expected, staining was not detectable in the lungs of Cavin1−/− mice (Fig. 1B).

**Figure 2:**
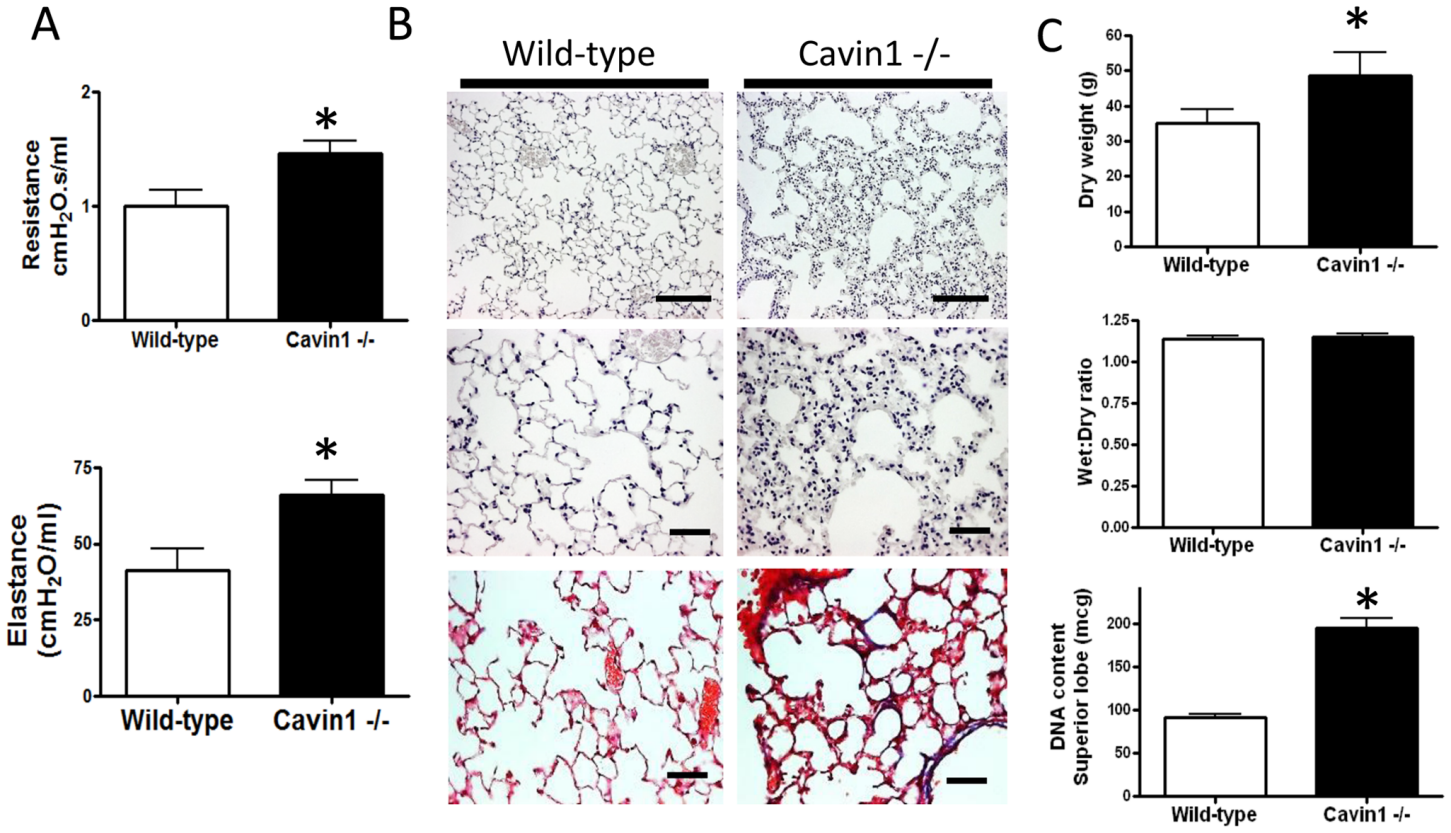
Cavin1 deficiency leads to physiological and structural changes to lung. A) Airway resistance and lung elastance were increased in lungs of Cavin1−/− mice (n = 6, both groups, 2 independent experiments). B) Low power images of H&E stained lungs (upper images) show architectural distortion in the lungs of Cavin1−/− mice. High power images (middle images) demonstrate hypercellularity (increased blue nuclei) in the distal lung in Cavin1−/− mice. Trichrome staining (lower images) demonstrates increased deposition of collagen (blue) in the lungs of Cavin1−/− mice. C) Dry weight (n = 5, each group, p<0.05) and DNA content (n = 10, each group, p<0.001) are increased in the lungs of Cavin1−/− mice. Statistically significant differences were not observed in lung wet-to-dry ratios from wild-type and Cavin1−/− mice (n = 5, each group).

Given the abundance of Cavin1 in lung, we investigated the functional role for this protein by performing detailed analyses of lungs from Cavin1−/− mice. Physiological measurements demonstrated increased airways resistance and lung elastance in Cavin1−/− mice (Fig. 2A), but the most dramatic finding came from histological analyses, which showed markedly altered distal lung morphology and significant thickening of the lung interstitium in Cavin1−/− mice (Fig. 2B - upper and middle images). Trichrome staining illustrated that lung interstitial thickening was associated with an increase in collagen deposition (Fig. 2B – lower images). Moreover, there were increased numbers of nuclei detected within the lung interstitium of Cavin1−/− mice after staining with hematoxylin (Fig. 2B – middle image), and this hypercellular phenotype was consistent with the observed increase in dry weight and DNA content in lungs of Cavin1−/− mice (Fig. 2C).

**Figure 3:**
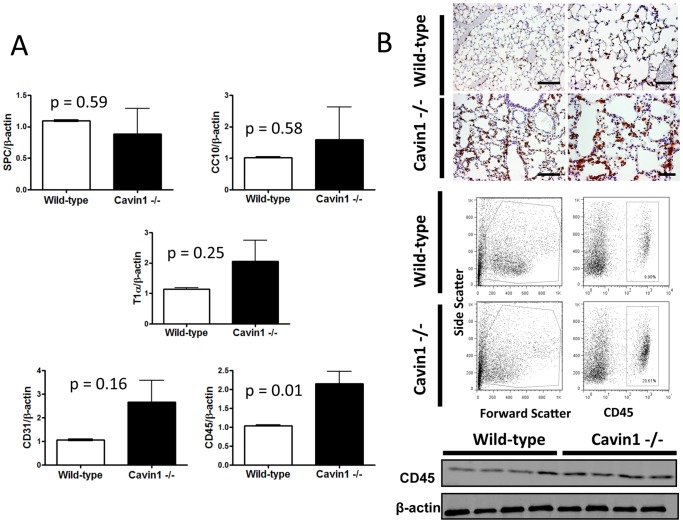
Leukocytes are increased in the lungs of Cavin1−/− mice. A) Transcript levels for structural and non-structural cells in lung of wild-type and Cavin1−/− mice. Transcript levels for the hematopoietic marker CD45 are increased in the lungs of Cavin1−/− mice. Statistically significant differences were not observed in transcript levels for SPC, CC-10, T1α, CD31 in the lungs of wild-type and Cavin1−/− mice (n = 5 or greater in all groups, two replicates). B) CD45-positive cells are increased in the lungs of Cavin1−/− mice. (Top) Immunohistochemistry staining for mouse CD45. Brown straining represents CD45+ expressing cells. (Middle) Flow cytometry analysis of CD45 stained lung digests from wild-type and Cavin1−/− mice (n = 6 wild-type and Cavin1−/− mice). Doublets, red blood cells and dead cells were excluded from analysis. (Bottom) Western blot analysis for CD45 in lungs from wild-type and Cavin1−/− mice.

To evaluate whether hypercellularity was attributable to increases in specific cell populations, quantitative RT-PCR was performed to screen for alterations in endothelial, epithelial and leukocyte-specific markers in the lungs of wild-type and Cavin1−/− mice (Fig. 3A). We did not identify any differences in transcript levels for the proximal epithelial gene CC-10 or the distal type II epithelial cell marker SP-C; however, non-significant increases were observed in levels of the endothelial marker CD31 and the distal type I epithelial marker T1α. Most notably, we found that transcript levels for the hematopoietic marker CD45 were significantly increased in lungs of Cavin1−/− mice.

**Figure 4:**
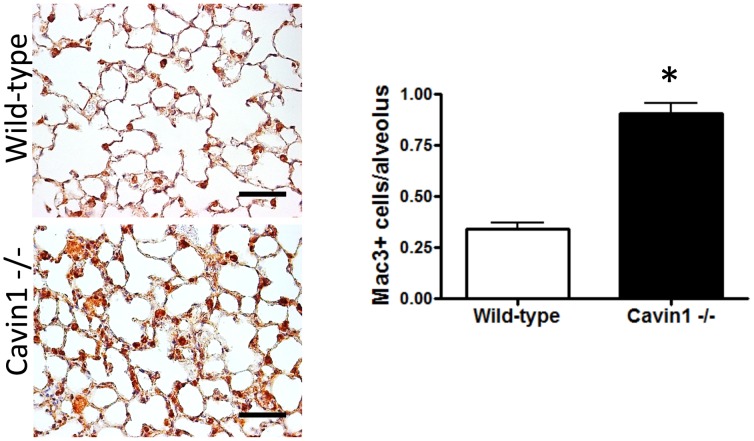
Macrophages are increased in the lungs of Cavin1−/− mice. (Left) Immunohistostaining for Mac3 in lungs of wild-type and Cavin1−/− mice. (Right) Morphometric data showing numbers of Mac3+ cells/alveolus in wild-type and Cavin1−/− mice (300 alveoli/per mouse, 5 mice per group, p<0.001).

Since differences in CD45 transcript levels were largely unexpected, we sought to confirm whether the number of leukocytes was in fact increased in the lungs of Cavin1−/− mice. Antibody staining directed against CD45 identified increased numbers of leukocytes in histological sections as did both flow cytometry (CD45+ cells in digested lungs were 15% +/− SEM 1.3% in wild-type mice and 27% +/−1.4% SEM in Cavin1−/− mice, p<0.05) and Western blot analysis from digested lungs of Cavin1−/− mice (Fig. 3B). Surprisingly, immunophenotyping in tissue sections did not identify differences in cells expressing T-cell (CD3), B-cell (B220), NK cells (NK1.1) or granulocyte (Gr-1) specific markers (data not shown). However, significant increases were observed in cells expressing the macrophage-marker Mac3 in lungs of Cavin1−/− mice (Fig. 4). This selective increase in Mac3+ cells was also associated with a 3-fold rise in macrophages in BAL fluid of Cavin1−/− mice (9.7+/−13 x10^4^ cells/lung wild-type versus 23.7+/−44 x10^4^ cells/lung Cavin1−/− mice, n = 5 each group). Importantly, similar increases were not observed in the number of mononuclear cells in the circulation of Cavin1−/− mice (3996+/−247 cells/mm^3^ wild-type versus 3899+/−436 cells/mm^3^ Cavin1−/− mice, n = 5 each group).

**Figure 5:**
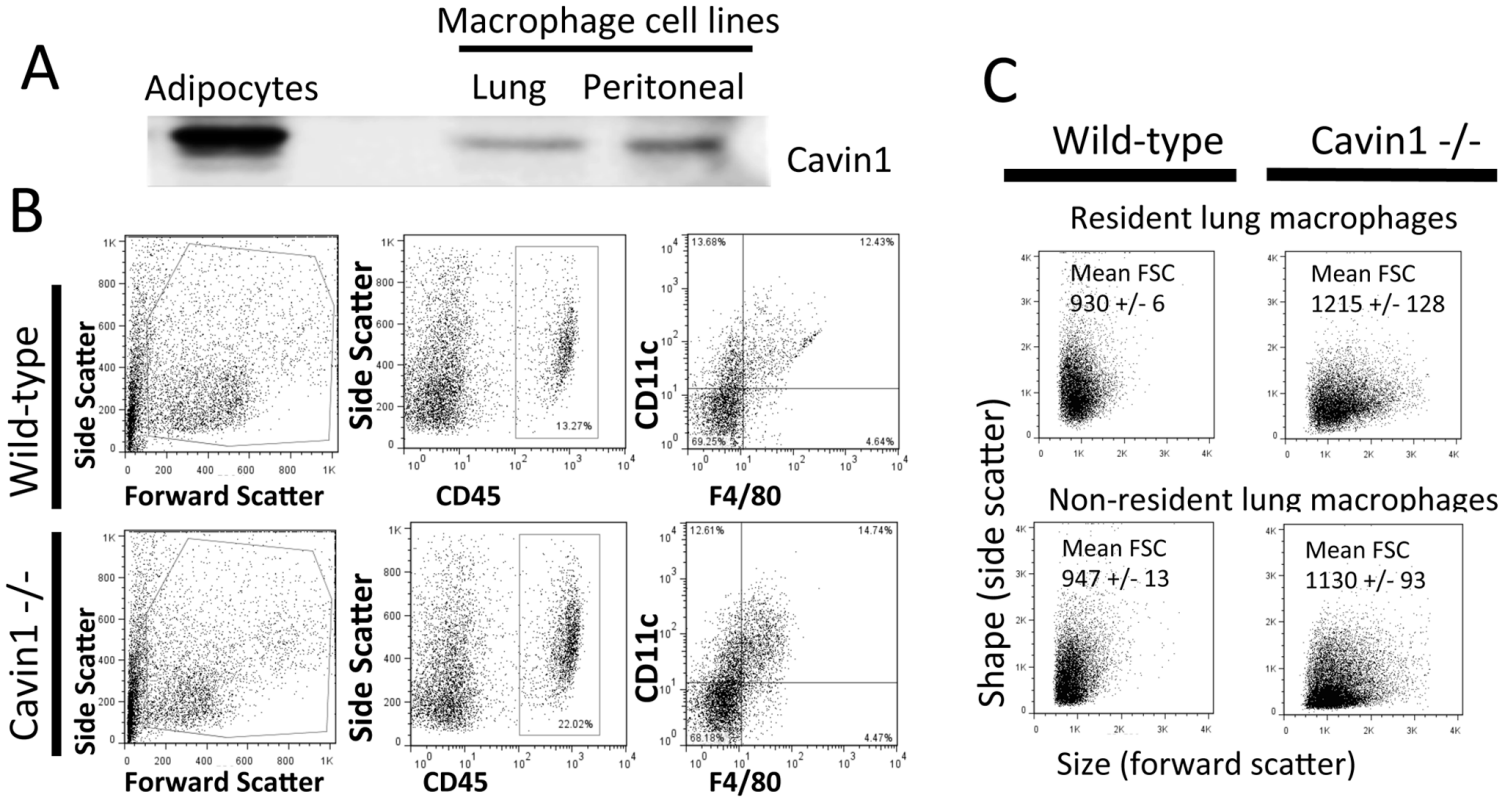
Morphological comparison of macrophages from lungs of wild-type and Cavin1−/− mice. A) Western blot analysis showed expression of Cavin1 in lung (MHS Cells) and peritoneal (RAW) macrophage cell lines. Relative concentrations were decreased when compared to Cavin1 levels in an adipocyte cell line (2 independent analyses). B) Flow cytometry analysis of lung digests from wild-type and Cavin1−/− mice after staining for F4/80 and CD11c (n = 3, both groups). Doublets, red blood cells and dead cells (propidium iodide positive) were excluded from analysis. C) Forward and side-scatter characteristics of resident (CD11c) and non-resident (CD11b+) macrophages in lung digests (n = 3 each group). Cell size (forward scatter) was significantly increased (p<0.05) in both resident and non-resident macrophages from Cavin1−/− mice.

After confirming Cavin1 transcript expression in freshly isolated alveolar macrophages (data not shown) and Cavin1 protein expression in multiple macrophage cell lines (Fig. 5A), we evaluated whether deficiency in Cavin1 was associated with qualitative differences in lung macrophages. Flow cytometry identified a trend toward increased F4/80+ CD11c+ cells in lung digests of Cavin1−/− mice, but this did not reach statistical significance (Fig. 5B). However, in separate studies, clear differences were observed in the morphological characteristics of macrophages recovered from lung digests during forward and side-scatter analysis. As shown in Figure 5C, resident CD11c+macrophages and non-resident CD11b+monocyte/macrophage populations from lung digests of Cavin1−/− mice were on average larger (forward scatter) when compared to those from wild-type mice.

**Figure 6:**
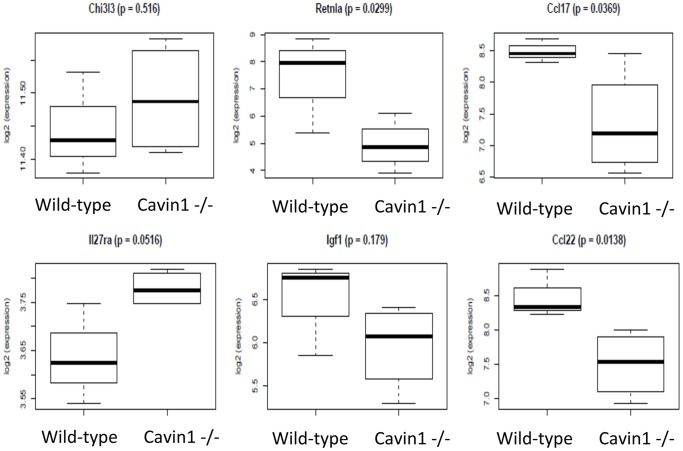
Expression of macrophage-specific genes associated with remodeling and repair. Gene transcripts that were statistically different in Cavin1−/− versus wild-type mice included Retnla (fold change −7.1, *p* = 0.030), Ccl17 (fold change −2.0, *p* = 0.037), Ccl22 (fold change −1.9, *p* = 0.014) but other genes in this category (IL27ra, Igf1 and Chi3l3) were not statistically different between groups.

In addition to changes in physical characteristics, microarray analysis demonstrated measurable differences in gene expression between alveolar macrophages (isolated by BAL) from wild-type and Cavin1−/− mice. The expression of 1393 genes was significantly (nominal *p*<0.05) altered by Cavin1 deficiency (more genes than would be expected by chance). Surprisingly, M1 and M2 gene transcripts were either expressed at low levels or did not change significantly (*p*>0.05) between wild-type and Cavin1−/− cells (data not shown); however, several macrophage-specific genes associated with remodeling and repair were significantly down-regulated in the Cavin 1−/− mice (Fig. 6) [33].

**Figure 7:**
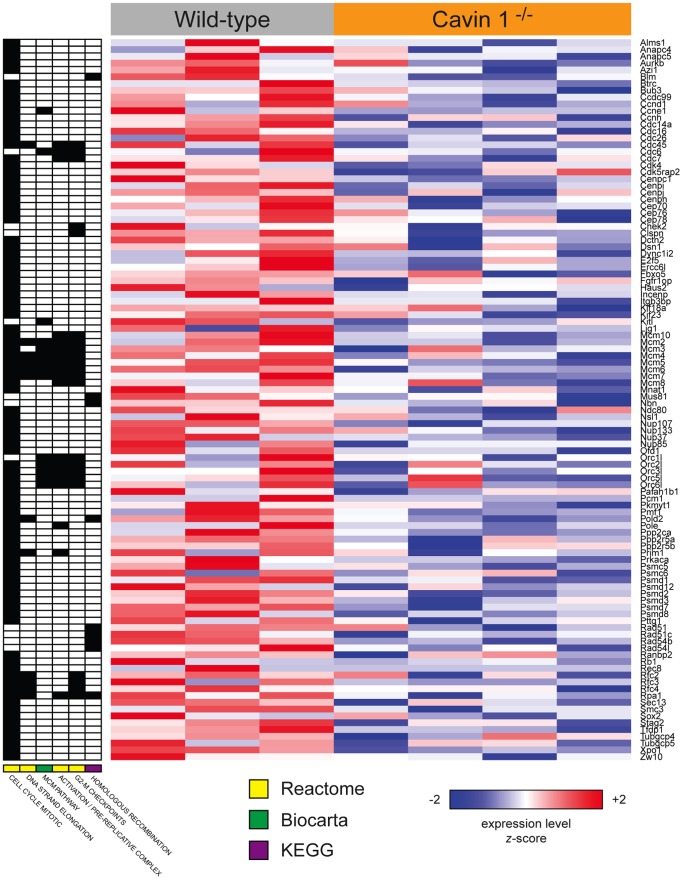
Gene Set Enrichment Analysis (GSEA). GSEA performed to identify Gene Ontology (GO) terms or Biocarta, KEGG or Reactome pathways that were differentially regulated between alveolar macrophages of wild-type and Cavin1−/− mice. Of the total 14 gene sets significantly over-represented (FDR-corrected *p*<0.05) in Cavin1-deficient mice six of these sets, which are represented in this figure, were related to proliferative processes, such as mitosis, DNA replication, and homologous recombination, and all were coordinately down-regulated in the Cavin1−/− mice.

Gene Set Enrichment Analysis (GSEA) was then performed to identify Gene Ontology (GO) terms or Biocarta, KEGG or Reactome pathways that were differentially regulated between alveolar macrophages of wild-type and Cavin1−/− mice. A total of 14 gene sets were significantly over-represented (FDR-corrected *p*<0.05) among genes that were up- or down-regulated in Cavin1-deficient mice (Table S1). Six of these sets were related to proliferative processes, such as mitosis, DNA replication, and homologous recombination, and were coordinately down-regulated in the Cavin1−/− mice (Fig. 7). Genes that were frequently represented among these gene sets included many key mediators of cell cycle control, especially the Mcm (minichromosome maintenance complex), Cdc (cell division cycle), Orc (origin recognition complex) and Rfc (replication factor C) family members (Fig. 7).

**Figure 8:**
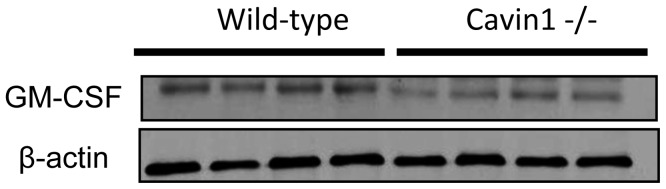
Western blot analysis for granulocyte-macrophage colony-stimulating factor (GM-CSF) in lungs from wild-type and Cavin1−/− mice (same polyvinylidene difluoride (PVDF) membrane used in **figure 3**.

Given that coordinate down-regulation of mitotic genes was observed in macrophages from Cavin1−/− mice, we evaluated whether these cells displayed any differences in proliferation relative to the wild-type macrophages. There was no difference in proliferation as measured by PCNA staining, and consistent with the microarray analysis, we detected significant decreases in both transcript (data not shown) and protein levels (Fig. 8) of the macrophage proliferation cytokine GM-CSF. Interestingly, these findings suggest that maturation and function of alveolar macrophages is likely to be impaired by GM-CSF deficiency in Cavin1−/− mice.

**Figure 9:**
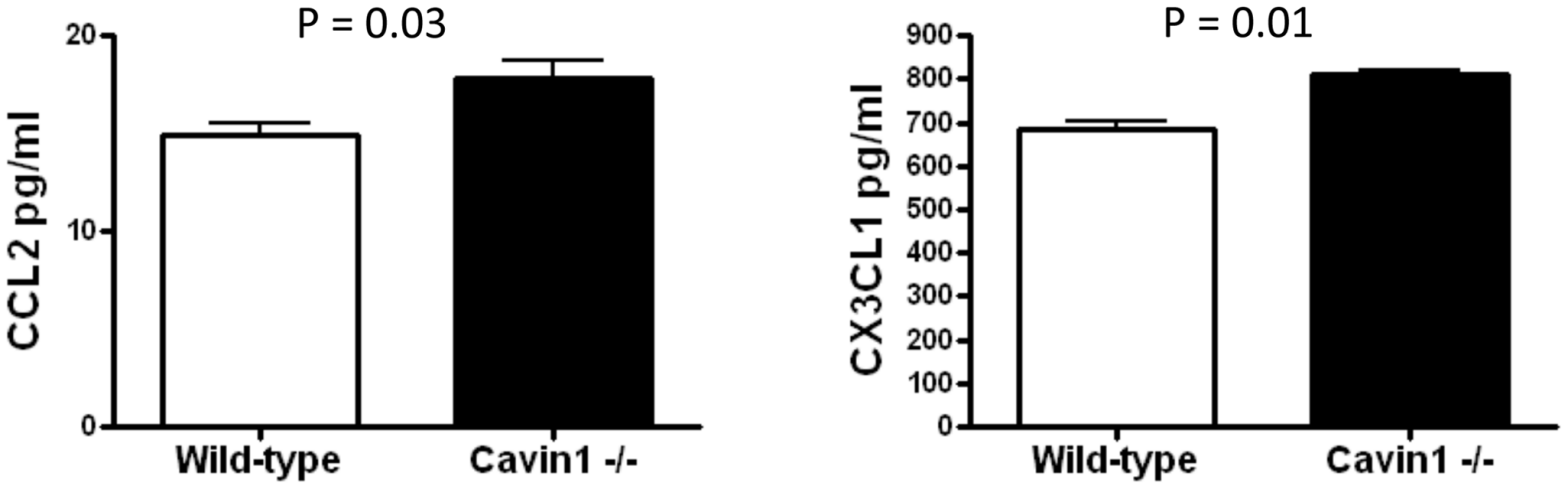
CCL2 and CX3CL1 are increased in the lungs of Cavin1−/− mice. Enzyme-linked immunosorbent assay for CCL2 and CX3CL1 in lungs of wild-type and Cavin1−/− mice (n = 4, each group, two replicates).

Since macrophage gene expression and PCNA labeling suggested that increased proliferation was unlikely to explain macrophage accumulation in Cavin1−/− mice, we sought to evaluate whether other mechanisms, such as increased recruitment may have contributed to these findings. Apoptotic cell debris is well-recognized as an important stimulus for macrophage recruitment, but TUNEL staining failed to detect evidence for increased apoptotic cell death in unchallenged lungs from Cavin1−/− mice (data not shown). However, modest but statistically significant increases were detected in the levels of macrophage chemotactic factors CCL2 and CX3CL1 in BAL fluid from Cavin1−/− mice (Fig. 9). Together, these findings suggest that increased recruitment may contribute, at least in small part, to macrophage accumulation in lungs of Cavin1−/− mice.

**Figure 10:**
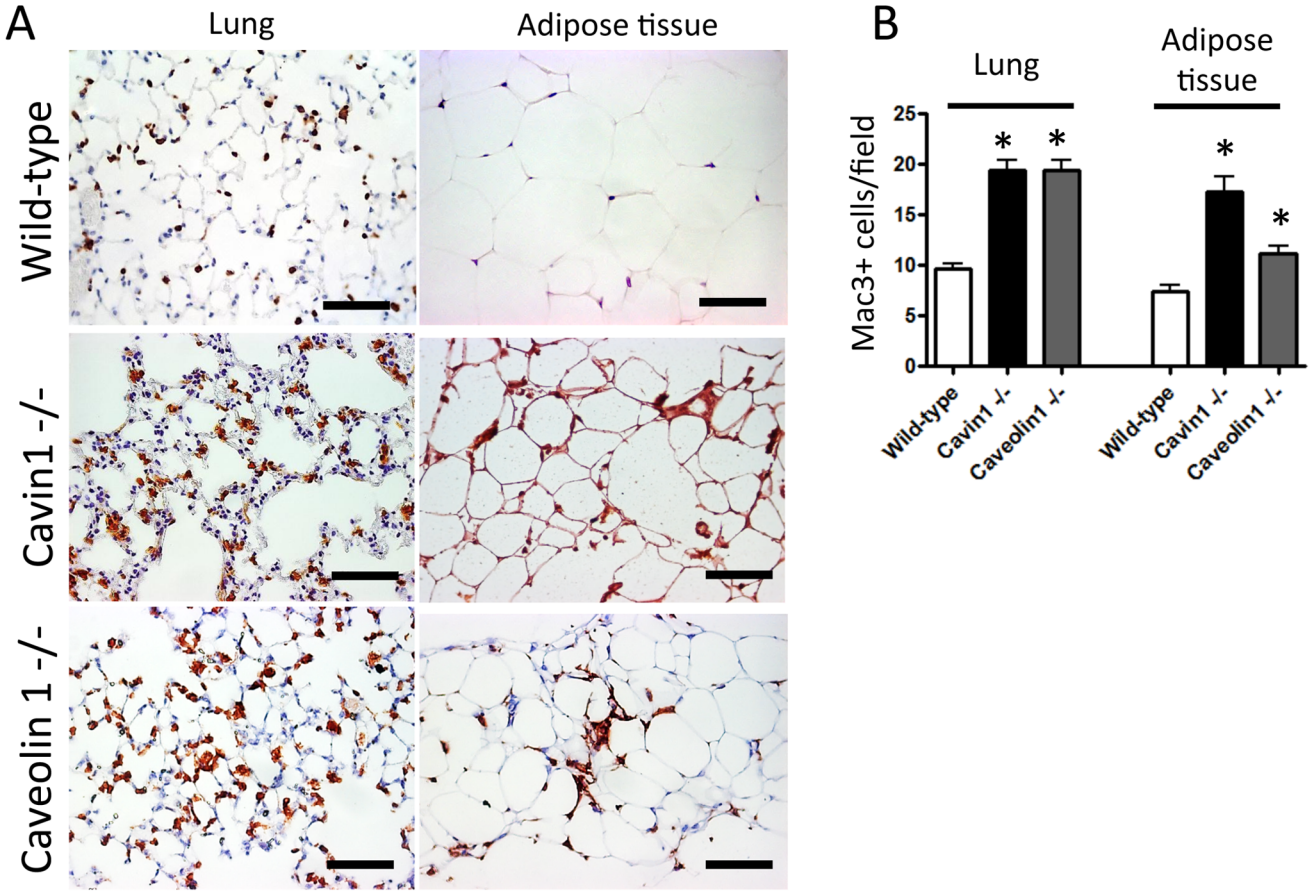
CD45 and Mac3 staining of tissues from wild-type, Cavin1−/− and Cav1−/− mice. A) Representative sections from CD45 stained lung and adipose tissue from wild-type, Cavin1−/− and Cav1−/− mice (n = 4, all groups). B) Results of morphometric analyses on Mac3 stained lung and adipose tissue sections from wild-type, Cavin1−/− and Cav1−/− mice (n = 4, all groups). Differences in Mac3+ cells were not observed in liver, kidney or skeletal muscle in either group (data not shown).

Finally, to evaluate whether alterations in macrophage homeostasis were specific to either the lung or to Cavin1 deficiency, immunostaining for leukocyte antigens CD45 and Mac3 was performed in multiple tissues from wild-type, Cavin1−/− and Cav1−/− mice. Increased staining for CD45+ and Mac3+ cells was observed in lung and adipose tissue (Fig. 10), but not in liver, kidney or skeletal muscle from knock-out mice (data not shown). These findings highlight the importance of Cavin1 and Cav1 in regulating macrophage homeostasis but suggest this property is restricted to selective tissues (e.g. lung and adipose tissue).

## Discussion

Caveolae are prominent morphological features of lung endothelium and epithelium that are important for respiratory function [34]. These properties are underscored by the dysfunctional phenotype of the Cav1 deficient mouse that lacks caveolae, shows thickening of the alveolar septa and respiratory functional defects [4]. Interestingly, the Cav2 deficient mouse, a protein thought of as the structural partner for Cav1 [35] exhibits a similar pulmonary phenotype as does the Cav1 deficient animal, but it shows no loss of caveolar structure [5]. With this context in mind, we examined, for the first time, the pulmonary phenotype of the Cavin1 knockout mouse and showed that Cavin1 is critical for regulating lung homeostasis and that its deficiency leads to altered structure and function of the lung. Most notable among these changes were physiological (e.g. increased elastance) and biochemical alterations (e.g. increased extracellular matrix) as well as marked hypercellularity. While collectively these pathological findings complement those described for lungs of Cav1 and Cav2−/− mice, unique to this investigation was the observation that leukocytes rather than structural cells contributed to lung hypercellularity [3]–[5].

In previous reports, hypercellularity in lungs of Cav1−/− mice was attributed to increases in Flk1+ “endothelial progenitors” [4]. Interestingly, upon detailed review of these publications, Flk1+ cells were noted to possess morphological characteristics atypical for endothelial cells (e.g. small and round) and found to reside in locations similar to the CD45+ cells in our study [4]. These findings, along with reports demonstrating low-level expression of Flk1 on macrophages, support the notion that at least a subset of “endothelial progenitors” may in fact be immune cells [36]–[38].

Detailed characterization of lung leukocytes in Cavin1−/− mice found that increased cellularity was largely attributable to macrophage infiltration, an event that has also been reported in adipose tissue of mice lacking caveolae [39]. However, along with changes in cell numbers, we also found that Cavin1 deficiency led to a significantly altered lung macrophage phenotype. This was most evident when comparing cell size during flow cytometry analysis, but other qualitative changes were readily apparent during gene expression profiling.

Despite identifying significant changes in macrophage gene expression, we found that macrophage expression profiles did not fit into classic M1 or M2 paradigms [33]. These findings are in contrast to a recent study showing up-regulation of M2-related genes in adipose-tissue derived macrophages from Cav1−/− mice [39]. These differences between studies are multi-factorial and likely relate to tissue, macrophage and structural protein related factors. Another notable finding from our microarray analysis was the observation that genes associated with repair and remodeling were down-regulated in macrophages from Cavin1−/− mice. Given that extracellular matrix deposition appeared to be increased in the lungs of Cavin1−/− mice, we speculate that down-regulation of these genes may be an adaptive response to the already remodeled architecture in lungs these mice. Moreover, these findings suggest that macrophages do not significantly contribute to baseline fibrosis in these mice.

The mechanisms leading to lung macrophage accumulation in Cavin1−/− mice cannot as yet be defined precisely. However, increased numbers of macrophages in the distal airspaces of Cavin1−/− mice indicated that cells were unlikely to be simply trapped in the thickened interstitium of the lung. Additionally, normal circulating numbers of mononuclear cells illustrated that systemic alterations in hematopoiesis were unlikely to have contributed significantly. Findings to suggest macrophage accumulation in Cavin1−/− mice was due to increased cellular recruitment were based on increased levels of macrophage chemotactic factors CCL2 and CX3CL1 in BAL fluid from Cavin1−/− mice. However, because increases in these chemokines were quite modest, it seems unlikely that this mechanism entirely explains the macrophage accumulation in lungs Cavin1−/− mice. With this in mind, we speculate that impaired egress could also play a role [40], [41] since leukocytes utilize caveolae on endothelial cell surfaces to enter and to exit tissues [42].

While numerous reports describe a role for caveolae in suppressing cell proliferation [3], [43], [44], the findings in our study (i.e. absence of PNCA labeling and down-regulation of cell cycle related genes) indicate that increased proliferation was not a major contributor to macrophage accumulation in Cavin1−/− mice. Although absence of proliferation was unexpected from Cavin1 deficiency, these findings are, however, consistent with published studies on macrophages, which indicate these cells do not proliferate in the lung under basal, non-stressed conditions [22].

Another important finding in this study was the observation that transcript and protein levels for GM-CSF were markedly decreased in lungs from Cavin1−/− mice. GM-CSF is a 23 kD glycoprotein that functions to promote monocyte growth and differentiation but acts in lung to regulate not only macrophage number and phenotype but also various critical functions such as surfactant phospholipid catabolism and the clearance of pathogens from the lung. [45]–[47] Although our study did not go on to evaluate whether GM-CSF replacement therapy restores macrophage number and phenotype in lungs from Cavin1−/− mice, or for that matter in adipose tissue, we speculate that its deficiency is likely to play a mechanistic role in these findings.

Finally, while the focus of this paper was to investigate cavin1’s role in the lung, it is worth emphasizing that alterations in macrophage homeostasis were not restricted to this tissue. The reasons for the selective increase in macrophages in lung and adipose tissue are not obvious, but it seems logical to assume that characteristics shared by these organs may explain the abnormal macrophage accumulation in these tissues. Notably, one feature shared by the lung and adipose tissue is their role in regulating lipid regulation. Adipose tissue stores and releases lipids in response to metabolic cues and lipid biosynthesis and clearance are critical for maintaining the functional characteristics of the lung’s surface lining fluid. Thus, we postulate, that altered lipid metabolism may be a factor promoting macrophage accumulation in lung and adipose tissue; a hypothesis supported by decreased GM-CSF levels in lungs from Cavin1−/− mice [45]–[47].

In conclusion, this study is the first to describe a role for Cavin1 in the lung. Our findings indicate that targeted deletion of Cavin1 leads to a complex lung phenotype that includes alterations in respiratory function as well as changes to lung macrophage number and phenotype. We believe these findings expand our understanding of the lung and implicate caveolar structural proteins in the regulation of lung macrophage homeostasis.

## Supporting Information

Table S1 **Gene sets with significant enrichment in genes whose expression is altered in Cavin 1−/− macrophages.** Gene Set Enrichment Analysis (GSEA) was performed on 1,625 gene sets from Gene Ontology, KEGG, Reactome, and Biocarta, and 14 of these gene sets were found to be significantly enriched (FDR q <0.05) among genes whose expression was up- or down-regulated in Cavin 1−/− versus wild-type macrophages.
(DOCX)
