# Supplementary material for: Cavin1; a Regulator of Lung Function and Macrophage Phenotype
Source: PLoS One. 2013 Apr 25;8(4):e62045. doi: 10.1371/journal.pone.0062045 (PMC3636273; doi:10.1371/journal.pone.0062045)
Supplement: Table S1 — Gene sets with significant enrichment in genes whose expression is altered in Cavin 1−/− macrophages. Gene Set Enrichment Analysis (GSEA) was performed on 1,625 gene sets from Gene Ontology, KEGG, Reactome, and Biocarta, and 14 of these gene sets were found to be significantly enriched (FDR q <0.05) among genes whose expression was up- or down-regulated in Cavin 1−/− versus wild-type macrophages. (DOCX) [file pone.0062045.s001.docx]

| **Category** | **Gene set** | **Number of genes** | **Enrichment Score (ES)** | **Normalized Enrichment Score (NES)** | **Nominal p** | **FDR q** |
| --- | --- | --- | --- | --- | --- | --- |
| Reactome | Electron transport chain | 57 | 0.52 | 2.12 | < 0.001 | 0.033 |
| KEGG | Vibrio cholerae infection | 46 | -0.53 | -1.92 | < 0.001 | 0.044 |
| Reactome | G2-M checkpoints | 41 | -0.53 | -1.92 | < 0.001 | 0.047 |
| Biocarta | MCM pathway | 18 | -0.66 | -1.93 | < 0.001 | 0.044 |
| GO Term | Oxidoreductase activity | 32 | -0.56 | -1.95 | < 0.001 | 0.039 |
| KEGG | Homologous recombination | 27 | -0.59 | -1.96 | < 0.001 | 0.036 |
| Reactome | Cell cycle, mitotic | 289 | -0.39 | -1.97 | < 0.001 | 0.036 |
| KEGG | Lysosome | 116 | -0.46 | -2.00 | < 0.001 | 0.026 |
| Reactome | DNA strand elongation | 30 | -0.60 | -2.01 | < 0.001 | 0.027 |
| Reactome | tRNA aminoacylation | 40 | -0.57 | -2.07 | < 0.001 | 0.011 |
| Reactome | Cytosolic tRNA aminoacylation | 23 | -0.67 | -2.09 | < 0.001 | 0.010 |
| Reactome | Activation of the pre-replicative complex | 29 | -0.63 | -2.12 | < 0.001 | 0.010 |
| GO Term | Oxidoreductase activity acting on the aldehyde or oxo group of donors | 21 | -0.76 | -2.35 | < 0.001 | 0.001 |
| GO Term | Oxidoreductase activity acting on the aldehyde or oxo group of donors, NAD or NADP as acceptor | 15 | -0.84 | -2.36 | < 0.001 | 0.001 |
